# Moral Distress, Professional Burnout, and Potential Staff Turnover in Intensive Care Nursing Practice in Latvia—Phase 1

**DOI:** 10.3390/ijerph22081261

**Published:** 2025-08-12

**Authors:** Olga Cerela-Boltunova, Inga Millere, Evija Nagle

**Affiliations:** 1 Department of Nursing and Midwifery, Riga Stradiņš University, LV-1067 Rīga, Latvia; 2 Department of Nursing and Midwifery, Faculty of Health and Sports Sciences, Riga Stradiņš University, LV-1067 Rīga, Latvia; 3 Department of Health Psychology and Pedagogy, Rīga Stradiņš University, LV-1009 Rīga, Latvia

**Keywords:** intensive care unit, moral distress, burnout, staff turnover, nurses, Latvia

## Abstract

Burnout and moral distress are increasingly recognized as critical challenges within healthcare systems, particularly in high-stress environments such as intensive care units (ICUs). This cross-sectional study investigates the prevalence and interrelationships of moral distress, burnout, and turnover intentions among ICU nurses in Latvia, a country facing significant nursing shortages and structural workforce challenges. A total of 155 ICU nurses completed validated instruments assessing moral distress, the three subscales of burnout (personal, work-related, and client-related), and intentions to leave the profession. The results indicate that 68.2% of respondents experienced moderate to high levels of moral distress, especially related to providing aggressive treatment contrary to clinical judgment. Burnout scores were highest in the personal and work-related dimensions, with emotional exhaustion strongly correlated with moral distress. Approximately 30% of participants reported active intentions to leave their positions. Regression and mediation analyses confirmed that moral distress significantly predicted both burnout and turnover intentions, with burnout partially mediating this relationship. These findings highlight urgent risks not only to nurse well-being but also to healthcare quality and sustainability. This study underscores the importance of systemic interventions, including structured workload assessment tools, psychological support, and ethical consultation services. The results contribute to the international literature and offer context-specific insights for workforce resilience in Eastern European health systems.

## 1. Introduction

The psycho-emotional well-being of intensive care unit (ICU) nurses has emerged as a pressing public health concern, given the high levels of psychological strain and professional responsibility inherent to this clinical setting [1]. Nurses daily confront life-threatening conditions, complex ethical decisions, and high patient acuity, which together create a uniquely stressful work environment [2]. These persistent stressors significantly elevate the risk of emotional exhaustion, moral distress, and ultimately, professional burnout [3].

Numerous studies worldwide have confirmed that ICU nurses experience disproportionately high levels of moral distress, emotional exhaustion, and professional burnout compared to nurses in other clinical settings [3,4,5,6]. Contributing factors include understaffing, ethical dilemmas, high patient acuity, low autonomy, and limited access to institutional support such as psychological counseling or ethical consultation services [4,6,7,8]. These stressors not only compromise the well-being of nurses but also reduce care quality and increase the likelihood of workforce attrition [9,10].

Importantly, moral distress and burnout are not isolated phenomena. Studies in diverse healthcare systems have identified a pathway linking unresolved ethical conflict (moral distress) to emotional exhaustion (burnout), which in turn increases nurses’ intentions to leave the profession [8,11,12,13]. This cycle can create staff shortages, reduced care quality, and increased financial strain on healthcare institutions [14]. In under-resourced and aging health systems like Latvia’s [15], such dynamics pose a serious threat to the sustainability of the healthcare system.

The relevance of burnout and moral distress extends beyond individual health outcomes [1,3,4,7,8,10,11]. Burnout contributes to high turnover rates, reduced quality of care, medical errors, and absenteeism [1,4,11,12]. The World Health Organization (WHO) and international nursing organizations have emphasized the public health risks posed by burnout in the healthcare workforce [16]. Therefore, addressing ICU nurse burnout and its contributing factors, including moral distress and intentions to leave, is crucial not only for personal well-being but also for institutional sustainability and the resilience of the healthcare system [4,10,12,17].

Despite the growing international literature, there is a lack of comprehensive and representative empirical data on this issue in Latvia. Most existing studies in the Latvian context are conceptual or narrative reviews [18,19], and they do not reflect the real-time prevalence, severity, or interrelationships of these phenomena among ICU nurses. One of the few empirical studies was conducted by Cerela-Boltunova et al. [19], which highlighted the need for more robust data by showing significant associations between moral distress and two burnout components (emotional exhaustion and depersonalization). However, due to methodological and sample limitations, generalizable conclusions remain elusive. This knowledge [19] gap is concerning given the broader implications for healthcare sustainability, patient safety, and workforce retention in Latvia. The lack of systematic, large-scale data hinders evidence-based policy planning, institutional reforms, and the development of support mechanisms tailored to ICU nurses in Latvia.

The Latvian healthcare system operates a tiered model of intensive care, with regional and university hospitals offering different levels of services [20]. Nurse-to-patient ratios often remain inadequate, ranging from 1:2 to 1:4, especially during night shifts and in lower-level ICUs [21]. Many nurses work extended hours, including 24 h shifts, and there is limited institutional support in the form of psychological or ethical consultation services [20]. Structured workload assessment tools such as the Nursing Activities Score (NAS) or the Therapeutic Intervention Scoring System-28 (TISS-28) are not widely implemented, leaving much of the workload unmonitored. These contextual stressors amplify the psychological burden on ICU nurses and increase the urgency for empirical assessment [22,23,24].

By employing validated and culturally adapted psychometric instruments in a multicenter cross-sectional design, this study aims to provide empirical evidence on the prevalence and interrelation of moral distress, professional burnout, and turnover intentions among ICU nurses in Latvia. Although they are localized, the results have broader implications for other post-Soviet, under-resourced healthcare systems. In doing so, the study not only addresses national knowledge gaps but also contributes to the global discourse on nurse well-being and the sustainability of the healthcare workforce.

Based on existing theory and international evidence, the following hypotheses are proposed:

- H1: Higher levels of moral distress will be significantly associated with higher levels of burnout (emotional exhaustion and depersonalization).
- H2: Burnout will be significantly associated with turnover intention.
- H3: Moral distress will be significantly associated with turnover intention, with burnout mediating this relationship.

## 2. Theoretical Framework

The current study is grounded in an integrative theoretical approach to understanding the psycho-emotional burden experienced by ICU nurses, specifically focusing on three interrelated constructs: moral distress, professional burnout, and turnover intentions. These constructs are not only prevalent in high-acuity clinical environments but also have significant implications for healthcare system resilience and patient safety. The theoretical framework draws on established psychological, ethical, and organizational models to explain the complex interplay of factors influencing ICU nurses’ well-being.

The concept of moral distress was first introduced by Andrew Jameton in 1984, who described it as a psychological state that arises when healthcare professionals know the ethically correct action to take but are unable to act due to institutional constraints, hierarchical structures, or systemic inadequacies [5]. Since then, moral distress has been widely examined, particularly in intensive care settings, where nurses often face ethically complex and emotionally charged decisions, such as continuing aggressive treatment with limited benefit or witnessing patient suffering due to policy-driven care limitations [17].

Repeated exposure to morally distressing situations contributes to what Epstein and Hamric later termed “moral residue”, a cumulative emotional burden that may heighten psychological strain over time [25]. Studies have confirmed that ICU nurses are particularly susceptible to moral distress due to their proximity to end-of-life decision-making, limited decision latitude, and ambiguous team communication [17,26,27,28]. Inadequate staffing, communication breakdowns, and rigid institutional norms exacerbate these stressors, creating ethical conflicts between professional values and daily practices [23,28].

Professional burnout, as conceptualized by Maslach and Jackson, is a multidimensional syndrome consisting of three key components: emotional exhaustion, depersonalization, and reduced personal accomplishment [13,29,30]. Among ICU nurses, these dimensions may manifest as chronic fatigue, emotional withdrawal, reduced empathy toward patients, and a diminished sense of purpose in clinical practice [22,31,32]. Burnout is especially prevalent in high-demand settings, where inadequate resource conditions are often observed, such as in Latvian ICUs, where nurse–patient ratios are high [22,29].

Burnout has been widely studied in connection with moral distress. Multiple empirical studies have demonstrated that moral distress is a strong predictor of emotional exhaustion, which in turn contributes to other facets of burnout [3,8,11,23,24]. The cumulative effect of unresolved moral conflict, chronic stress, and professional frustration may erode nurses’ psychological resilience and professional engagement [3,11,24].

The third focal construct is turnover intention, defined as the conscious consideration or planning to leave one’s current job or profession altogether. Turnover intention is not only a reflection of individual dissatisfaction but also an important organizational indicator of impending staff attrition [22]. In ICU settings, turnover intentions are often associated with burnout, low job satisfaction, and repeated exposure to moral dilemmas [33]. For example, studies have shown that approximately 37% of ICU nurses have considered resigning due to unresolved ethical conflicts [34] and that burnout may fully mediate the relationship between moral distress and turnover intention [19,22].

Moral distress and burnout both contribute significantly to turnover intentions. Studies suggest that burnout may act as a mediator, whereby the experience of moral distress leads to emotional exhaustion, which then fosters the desire to leave [22,23,24]. This mediating pathway highlights the importance of addressing intermediate psychological processes when designing retention strategies. High turnover further disrupts continuity of care, increases the burden on remaining staff, and undermines institutional effectiveness, creating a self-perpetuating cycle of stress and attrition [14,22].

To comprehensively address the interplay among these variables, this study draws upon three complementary theoretical models:

- Maslach’s burnout theory explains the psychological progression from chronic stress to emotional exhaustion, depersonalization, and reduced professional efficacy [29].
- Jameton’s Concept of Moral Distress provides an ethical framework for understanding how systemic constraints impair moral agency and contribute to emotional strain [25].
- The Job Demands–Resources (JD-R) Model situates burnout and turnover intention as outcomes of disproportionate job demands (e.g., workload, emotional labor) and insufficient resources (e.g., autonomy, support), making it particularly suitable for under-resourced settings like Latvian ICUs [30].

In addition, Karasek’s Demand–Control Model reinforces this framework by positing that high psychological demands, combined with low control over one’s work, exacerbate job strain and burnout, particularly in hierarchical healthcare environments [35]. Together, these models provide a robust framework for examining how moral distress (ethical strain) and burnout (emotional strain) interact to shape turnover intention (behavioral response).

Despite the extensive international recognition of moral distress and burnout as critical issues in intensive care settings, empirical research on these phenomena remains disproportionately focused on high-resource Western healthcare systems, such as those in the United States, Canada, and Western Europe. These studies consistently demonstrate that ICU nurses face high levels of psychological and ethical strain, leading to negative outcomes for staff well-being and patient safety [1,3,4,7,8,10,11,12,17]. However, in lower-resource or transitional healthcare systems, such as Latvia and other post-Soviet countries, the lack of comprehensive empirical studies creates a significant gap in both academic understanding and policy development. In Latvia, the available literature on ICU nurse well-being is scarce and largely limited to conceptual reviews [19,20] or isolated pilot projects. For example, the national strategy on healthcare workforce sustainability acknowledges increasing psychological risks and burnout among nurses but offers limited evidence-based interventions due to insufficient data [15,21]. This scarcity of research underscores the urgent need for localized empirical studies that apply validated instruments and robust theoretical models to explore the interconnections among moral distress, burnout, and turnover. Such research is not only essential for aligning Latvia with international best practices but also for informing context-specific strategies that reflect the structural realities and systemic constraints of the national healthcare system.

## 3. Materials and Methods

This study is part of a larger doctoral research project titled “Moral distress, professional burnout, workload and potential staff turnover in Intensive Care Nursing practice”, which focuses on understanding and reducing the psycho-emotional burden among ICU nurses in Latvia.

### 3.1. Study Design, Participants, and Sampling Strategy

This study was designed as a quantitative, multi-center cross-sectional study with the aim of assessing the psychosocial status of ICU nurses, focusing on three key indicators: moral distress, professional burnout, and the risk of potential staff turnover. Between 1 February and 1 May 2025, ICU nurses from multiple Latvian hospitals were invited to complete a standardized electronic questionnaire.

ICU nurses who met the following criteria were included in this study: registered medical practitioners with a valid professional certificate actively working in an ICU in one of the four administrative regions of Latvia—Rīga, Kurzeme, Vidzeme, or Zemgale—providing direct patient care in an intensive care setting for more than 3 months, who gave informed consent to participate in the study. Nurses who worked only in administrative or educational roles or were on long-term leave (e.g., maternity leave) were not included.

The exact number of ICU nurses in Latvia is not centrally registered, as workforce statistics vary across institutions and regions. However, national estimates suggest that the total ICU nursing workforce ranges between 500 and 600 individuals. To determine the required sample size, an online Sample Size Calculator was used. Assuming an unknown population size, the recommended minimum sample size was calculated to be 96 participants. To ensure sufficient statistical power and account for potential dropouts or incomplete responses, the target sample size was increased to at least 100.

A convenience sampling strategy was used. ICU nurses were recruited through internal communications, which were facilitated by ICU department managers and professional nursing associations. Study information was distributed via institutional email, and printed notices were posted in staff areas.

The final sample consisted of 155 ICU nurses, of whom 96.1% were women, aged between 21 and 63 years (mean = 17.9 years of professional experience). Although it was not designed as a prevalence study in the strict epidemiological sense, the sample exceeds the minimum threshold for correlational and regression analyses, thus supporting this study’s inferential goals.

The questionnaires were administered via Google Forms, ensuring anonymity, security, and data integrity. Only nurses currently providing direct patient care in the ICU were included. Participants were instructed not to consult others while completing the questionnaire to minimize bias.

This study was conducted in accordance with generally accepted principles of research ethics and the guidelines set out in the Declaration of Helsinki [36]. Prior to the commencement of data collection, appropriate permission was obtained from the Ethics Committee of Rīga Stradiņš University under protocol number 2-PĒK-4/416/2023, dated 9 May 2023.

Participation in this study was completely voluntary and anonymous. Before completing the survey, all respondents were provided with information about the purpose of the study, the approximate time required to complete it (20–25 min), as well as their right to refuse participation at any time without negative consequences. By completing the electronic survey, respondents gave their implicit informed consent to participate in this study. No personally identifiable data were collected.

All collected data were stored in secure, password-protected electronic databases accessible only to the principal investigator and authorized research team members. Analyses were conducted on aggregated data to ensure that no individual participants or institutions could be identified. No physical or psychological risks to participants were anticipated or reported.

In addition to obtaining ethical approval, formal institutional permissions were obtained from each participating hospital to access context-specific data such as ICU type, staff schedules, and general patient load. No patient-level clinical data were accessed or analyzed. To protect the anonymity of participating institutions, hospital-specific data (e.g., workload structure or staffing ratios) are not publicly disclosed in this manuscript. Given the relatively small number of ICUs in Latvia and the potential for indirect identification, disaggregated data by hospital will be made available upon reasonable request, subject to additional confidentiality agreements. This decision aligns with the overarching objective of the larger doctoral research project, which prioritizes institutional anonymity alongside scientific transparency.

### 3.2. Measures and Psychometric Properties

This study employed three validated instruments adapted for use in Latvia [37,38,39,40,41,42]. Moral distress was measured using the Latvian-translated version of the Measure of Moral Distress for Healthcare Professionals (MMD-HP) [37,38], originally developed by Epstein et al. [37,41]. A conscious and theoretically grounded decision was made to independently evaluate the frequency and intensity dimensions of moral distress.

Burnout was assessed using the Copenhagen Burnout Inventory (CBI) [38], which includes three subscales: personal burnout, work-related burnout, and client-related burnout [39,40].

Turnover intentions were measured using the Anticipated Turnover Scale (ATS), which is a 12-item scale [41].

The selection of sociodemographic variables as potential predictors of moral distress, professional burnout, and turnover intention is grounded in prior theoretical and empirical research. Studies have consistently shown that nurse-related characteristics, such as age, length of service, type of ICU, and shift schedule, are associated with variations in emotional well-being, ethical stress, and professional sustainability in intensive care settings [3,7,10,11,12,17,24].

These sociodemographic and contextual factors are therefore included as independent variables in the present study to explore their potential predictive role and to identify risk profiles that may inform targeted interventions.

All instruments demonstrated acceptable to excellent internal consistency in this sample, with Cronbach’s alpha values ranging from 0.739 to 0.976 (see Supplementary Materials for full psychometric tables).

Given the use of self-reported data and a cross-sectional design, we assessed the potential presence of common method bias (CMB) using Harman’s single-factor test, which is one of the most widely applied diagnostic tools in behavioral research. An exploratory factor analysis (EFA) was conducted on all items from the main psychometric instruments using an unrotated principal component analysis (PCA) in SPSS. The results indicated that the first factor accounted for 27.83% of the total variance, which is well below the critical threshold of 40%, suggesting that common method bias is not a major concern in this study. These results, together with the procedural remedies applied (e.g., anonymity, neutral item wording, item separation), provide sufficient evidence that CMB does not significantly threaten the validity of the findings.

### 3.3. Data Analysis

In this study, the primary dependent variables were (1) moral distress, (2) professional burnout, and (3) turnover intention, which was measured using the ATS. The independent variables included a range of sociodemographic and contextual characteristics (e.g., age, length of service, ICU type, number of jobs, and work schedule). No formal control variables were introduced in this study phase; however, group comparisons and multivariate models considered key sociodemographic variables as covariates.

Data analysis was conducted using IBM SPSS Statistics version 28 (IBM Corp., Armonk, NY, USA). Frequency tables were used for categorical variables, while mean, standard deviation (SD), minimum (min), and maximum (max) values were calculated for continuous variables.

The internal consistency of the applied psychometric instruments was evaluated using Cronbach’s alpha coefficients, and item-total correlation analyses were used to identify any problematic items.

To assess associations between continuous variables, correlation analysis was performed. Pearson’s correlation coefficient (r) was used when the variables were normally distributed, and Spearman’s rho (ρ) was applied for ordinal or non-normally distributed variables. Normality was assessed through Shapiro–Wilk tests and visual inspection of Q-Q plots. Correlation coefficients were interpreted using Cohen’s guidelines.

To examine group differences, the following statistical tests were applied: The independent samples *t*-test (parametric) and Mann–Whitney U test (non-parametric) were used for two-group comparisons. The one-way ANOVA and Kruskal–Wallis tests were used for comparisons across more than two groups (e.g., education levels, shift types). Tukey’s Honestly Significant Difference (HSD) test was used post hoc to detect specific group differences following significant ANOVA results.

Regression analysis was employed to identify significant predictors of moral distress, burnout, and turnover intention. Linear regression models were constructed for continuous dependent variables (e.g., burnout subscale scores). Binary logistic regression was used for dichotomous outcomes (e.g., considering leaving the job: yes/no).

To test the mediation hypothesis (H3), a simple mediation model (Model 4) was conducted using the PROCESS macro (version 4.1) by Andrew F. Hayes [43], with 5000 bootstrap samples. In this model, burnout was specified as the mediator between moral distress (predictor) and turnover intention (outcome). The indirect effect was interpreted as statistically significant when the 95% CI did not include zero.

All analyses assumed a two-tailed significance level of *p* < 0.05 unless otherwise specified. Results with *p* < 0.01 were reported as highly significant.

Finally, missing data were handled through listwise deletion in cases in which incomplete responses affected less than 10% of the total items. No extreme outliers (defined as z-scores ±3 SD) were identified. All analyses were based on the final complete sample of *n* = 155.

## 4. Results

### 4.1. Socio-Demographic Results of Respondents

As part of the study, all respondents were given a structured socio-demographic questionnaire aimed at obtaining general information about ICU nurses in Latvia. A total of 155 ICU nurses participated in the study. The majority of respondents were women (96.1%). Most nurses were employed in the Riga Region (79.4%). Regarding marital status, 50.3% reported being married.

In terms of workload, 50.3% reported working full time (40–50 h per week), while 14.8% worked 50–60 h per week and 7.7% worked more than 60 h per week. A total of 56.8% reported having one job, while 38.1% indicated working in two jobs.

Regarding turnover intentions, 50% of respondents had considered leaving their job due to moral distress, and 25.2% were currently considering it. More than 90% of nurses indicated that preventive measures are needed to reduce moral distress and burnout.

A detailed breakdown of the socio-demographic variables is provided in Supplementary Materials.

### 4.2. Moral Distress Scale Results

The overall Cronbach’s alpha for the MMD-HP scale was 0.938, indicating very high reliability. The mean values for individual questions ranged from 0.57 to 2.35, indicating that certain situations (e.g., questions 5, 10, 16, and 19) are experienced significantly more often than others. This leads to the conclusion that certain professional conflicts and dilemmas are a regular reality for nurses, while other situations (e.g., questions 6, 12, and 26) are less common.

The highest mean values were observed in questions 5 (M = 2.35), 16 (M = 2.18), 10 (M = 2.11), and 19 (M = 2.26). These items reflect clinically and emotionally complex situations in which nurses find themselves at the crossroads of ethical pressure or conflicting decisions. These questions can serve as key indicators for risk assessment if the scale is to be used in clinical practice.

The corrected item-total correlation scores are above 0.5 in most cases, indicating good coherence between items and the overall scale. The highest correlation with the overall scale was for question 24 (r = 0.768), and the lowest was for question 10 (r = 0.160), suggesting that this question is structurally or substantively inconsistent with the other items.

Similarly, the “Cronbach’s Alpha if Item Deleted” values for all items range from 0.933 to 0.943, indicating that the deletion of any item would not have a significant positive impact on the overall reliability of the scale. This further confirms the internal stability and psychometric quality of the scale. Table 1 presents the top five items with the highest item-total correlations and mean frequency scores, highlighting those most relevant to ICU nurses’ moral distress in the Latvian context.

A full item-level breakdown for all 27 items, including descriptive and psychometric statistics, is provided in Supplementary Materials.

Overall, the results of the “frequency” dimension show that Latvian ICU nurses encounter morally difficult situations relatively frequently in their daily work, and this frequency can be accurately assessed by the tool used. This scale is not only statistically valid but also clinically informative, allowing for the identification of the most common sources of moral distress, which can serve as a basis for the targeted development of preventive mechanisms.

In addition to frequency, the second part of the moral distress scale assesses the emotional intensity that nurses feel when they are in certain professional and ethical situations. The Cronbach’s alpha of the scale was 0.976, indicating excellent internal consistency. The overall mean score of the scale was M = 94.30, with a standard deviation SD = 40.03 and standard error SE = 1.61, while the spread of scores ranged from 0 to 97, indicating a very wide range of emotional responses among respondents. This shows that some nurses experience very low emotional impact, while for others, the situations cause significant psychological stress.

The mean values of the items ranged from 1.54 (question 21) to 2.63 (question 16). Similar to the frequency dimension, some situations (e.g., questions 5, 8, 9, 14, and 24–27) were assessed as particularly emotionally influential, with average scores above 2.4 points. These questions reflect ethically complex or emotionally stressful clinical situations, such as acting contrary to one’s values, limited options for action, or conflict situations with doctors or management.

The corrected item-total correlation was higher than 0.54 in all cases, exceeding 0.80 in most cases, indicating good coherence between items and the overall scale. The highest correlations with the total score were observed for questions 24–26 (e.g., r = 0.855 and higher), which may indicate their particular importance in determining the emotional intensity of distress.

Similarly, the “Cronbach’s Alpha if Item Deleted” values remain very high in all cases (between 0.975 and 0.976), confirming that the deletion of any item would not provide a significant benefit to overall reliability. This indicates the stability of the scale and no need for content adjustments from a statistical point of view. Table 2 presents the top five items with the highest item-total correlations and mean intensity scores, highlighting those most relevant to ICU nurses’ moral distress in the Latvian context.

A full item-level breakdown for all 27 items, including descriptive and psychometric statistics, is provided in Supplementary Materials.

These results confirm that the emotional intensity of moral distress among Latvian ICU nurses is clearly felt and varies across different situations. The high internal reliability of the scale and high correlation scores allow this dimension to be used both for identifying individual distress patterns and for developing policy and preventive measures, such as targeted support programs, particularly in distressing clinical situations.

### 4.3. Copenhagen Burnout Inventory Results

To assess the phenomenon of burnout among intensive care nurses, a Latvian adaptation of the CBI [41] was used, consisting of three subscales: personal-related burnout (PRB), work-related burnout (WRB), and client-related burnout (CRB) dimensions. The overall internal consistency (Cronbach’s alpha) was 0.928, indicating excellent internal reliability for the entire set of burnout scales. Table 3 presents the top five items with the highest mean scores, illustrating the most frequently experienced burnout symptoms among respondents. The full item-level psychometric overview for all 19 items is available in Supplementary Materials.

Total CBI scores across the scale were 37.59 out of a possible 76 points, or approximately 49.5% on a percentage scale, indicating a moderate level of burnout among ICU nurses in Latvia. This maximum value of 76 points represents the raw total score across all items. It is important to note that while some studies present CBI scores as percentages based on item-level transformations to a 0–100 scale, in this study, we opted for raw total scores to maintain comparability with our instrument’s internal structure. For interpretability, this raw score corresponds to an approximate average of 2.0 points per item on the original 1–5 Likert scale, which suggests a moderate burnout level. This rate is dangerously close to the 50% threshold, which most studies in the literature [43,44] identify as the point at which burnout starts to manifest in real clinical and professional symptoms (e.g., demotivation, errors at work, intention to change or leave job). The SD of the entire scale was 11.945, and the SE was 0.959. The minimum score obtained was 10, while the maximum was 64, indicating a wide range of experience among respondents with regard to burnout symptoms.

The average score for PRB subscale was 2.35, which corresponds to approximately 58.75% of the maximum burnout potential and indicates a moderately high level of personal burnout. Cronbach’s alpha for this subscale is 0.894, indicating high internal consistency. This means that nurses experience fatigue, exhaustion, and reduced motivation in relation to their professional selves moderately often. This level exceeds the 50% threshold, which suggests that it is clinically and organizationally significant and requires preventive monitoring.

The average WRB score was 2.20, corresponding to a percentage of 55%. The reliability coefficient for this subscale was 0.755, which is considered acceptable. This result can be interpreted as a moderate degree of burnout, mainly related to the work environment, prolonged overload, lack of management support or inefficient work organization. Given that there were also questions with very high standard deviations, there is a high degree of individual variability, indicating unequal experiences among nurses.

The mean CRB was 1.63, corresponding to a burnout rate of 40.75%. The reliability of this subscale was again 0.894, which is considered high. This is the lowest indicator among all three dimensions. This result indicates that emotional exhaustion in relation to patients is relatively low, and most nurses are able to maintain empathy and professional distance even in difficult situations. However, some questions (e.g., question 17 had a score of 2.19) also show increased emotional strain on some staff.

All three CBI dimensions have burnout rates above 40%, while two, PRB and WRB, have burnout rates above 50%, indicating the presence of systemic psychological overload. These results are considered moderately high and confirm the need to introduce targeted preventive measures at both the organizational and professional support levels. Since the CBI subscales have a very good psychometric structure, these data are considered reliable and interpretable for both research and management purposes.

### 4.4. Potential Staff Turnover Results

In this phase of the study, a 12-item scale was used to assess the potential for ICU nurses to leave their current job. The overall mean of the scale was M = 42.60, with SD = 8.378 and SE = 0.672. Scores ranged widely from 14 to 64, indicating substantial variability among respondents in their intention to remain in their jobs. This distribution shows that some nurses feel strongly attached to their jobs, while others have a strong desire to change jobs.

However, Cronbach’s alpha was only 0.342, indicating poor reliability and suggesting that the items most likely do not measure a single construct or contain several psychologically distinct dimensions. As a result, it is currently not advisable to interpret the scale as a single aggregate index; rather, it should be analyzed by items or structured into subgroups.

The mean values ranged from 1.80 (question 12) to 4.67 (question 7), indicating the highest levels of agreement with statements such as “I have no intention of leaving this job” (question 7) or “I feel that I belong to my work team” (question 1). This reflects a positive sense of professional belonging, but several items measuring items directly addressing intent to leave showed lower values, suggesting that some nurses are considering changing jobs.

The low corrected item-total correlation values (only one item exceeds the 0.3 threshold) and negative correlations of some items with the total scale indicate that some questions are not internally consistent with the rest of the content. Question 6 (I intend to stay with this organization as long as possible) and question 11 (Changing jobs is not important to me at the moment) may be too general or conceptually inconsistent with the others, which focus on action or intention. Table 4 displays the five highest scoring items by mean values, which reflect the strongest turnover-related tendencies among respondents. The complete item-level analysis is presented in Supplementary Materials.

Despite the low reliability, the data show that some ICU nurses have a strong desire to leave their job, but the majority of respondents report high levels of attachment to their team and work environment, which can serve as a protective factor. The items are phrased in different directions (some are statements about leaving, others about staying) and the responses were mixed, with some showing positive commitment and others revealing high ambivalence in theoretically or empirically supported dimensions. In future studies, this scale should be re-evaluated using EFA, and reformulation or division into separate subscales should be considered in order to achieve a clearer psychometric structure.

### 4.5. Correlation Results of the Study

To identify factors that statistically significantly predict the level of moral distress among ICU nurses, a multiple linear regression analysis was performed in the study. The total score on the moral distress scale was used as the dependent variable, while the independent variables included various socio-demographic, professional and subjective experience indicators (21 variables). This approach allowed us to assess which of the variables significantly influence distress levels while controlling for the influence of other factors.

To determine which socio-demographic and professional factors most significantly predict the level of moral distress among intensive care nurses, a linear regression analysis was performed, with the MMD-HP (total moral distress index) as the dependent variable, while 21 independent variables included job profile, workload, age, gender, region, education, intention to leave the job, and other aspects.

The overall regression model was statistically significant (F(21,133) = 3.234; *p* < 0.001), indicating that at least one of the independent variables included is significantly related to the level of moral distress. The coefficient of determination R^2^ = 0.338 (R = 0.581) indicates that the model explains 33.8% of the total variance in moral distress. Although this value is not very high, it clearly shows that part of the variability in distress is related to both the professional context and personal factors of nurses.

Of all the independent variables, only one was statistically significant (*p* < 0.05): “Have you ever left or thought about leaving clinical work because of moral distress?”, with a value of −0.317 (*p* < 0.00). This variable shows a negative and statistically significant association with MMD. This means that respondents who have considered or already left their jobs due to moral distress report a significantly higher level of current distress. This suggests a possible cumulative or chronic effect, in which a previous experience of distress increases sensitivity to new ethical and professional challenges. Two variables showed a trend toward statistical significance in the association between the level of moral distress and “Subjective perception of workload in the last month”, with a value of β = 0.132 (*p* = 0.099). The trend in these variables suggests that a higher subjective sense of overload may be associated with a higher level of moral distress. Although statistical significance has not been reached, this variable should still be considered a clinically relevant risk factor. The second variable, “Level of education”, was β = 0.184 (*p* = 0.043). Nurses with higher education levels may have an increased perception of distress, possibly due to higher ethical awareness, a more critical attitude toward systemic barriers, or a greater desire for professional autonomy.

High Variance Inflation Factor (VIF) values were observed for several variables. TISS-28 variables exhibited values of VIF ≈ 38, and evaluation of preventive measures exhibited values of VIF > 3.5. This suggests possible multicollinearity, particularly between questions on work organization, work supervision, and preventive measures. It would therefore be advisable to analyze these variables separately or reduce model complexity by selecting the most relevant variables based on theoretical significance or pre-regression analysis of correlations.

To identify the significant factors influencing the level of professional burnout among ICU nurses, a multivariate linear regression analysis was performed with CBI scores as the dependent variable. The independent variables included socio-demographic data, factors of the professional environment, and experiences and beliefs related to moral distress. The overall model quality is R = 0.695, R^2^ = 0.483, which means that the model explains 48.3% of the CBI variability. The ANOVA result is as follows: F (21,133) = 5.910 (*p* < 0.001), indicating the overall statistical significance of the model. Four independent variables showed a statistically significant impact on burnout rates. See Table 5.

The marital status of respondents is positively correlated with CBI results, indicating a difference in burnout levels between single and married respondents. Those who have considered leaving their job also report higher levels of burnout. The factor of “Currently considering leaving due to moral distress” was a statistically significant negative predictor (β = −0.387, *p* < 0.001).

This factor correlates even more strongly with burnout indicators, highlighting emotional overload and the mismatch between the healthcare system and nurses’ professional values. Factor 4 is as follows: “Have you ever completed the moral distress scale?” (β = −0.241, *p* = 0.002). This may indicate a greater awareness of professional risks or people with an already higher risk profile.

Multiple linear regression analysis was performed to investigate which factors are statistically significant predictors of potential staff turnover among ICU nurses. Overall, the model showed good fit (R^2^ = 0.338), explaining 33.8% of the variance in potential staff turnover (adjusted R^2^ = 0.234; F (21,133) = 3.239; *p* < 0.001). Of the 21 variables included, four predictors were statistically significant, indicating a notable impact on nurses’ intentions to change jobs. The results are shown in Table 6.

The negative beta coefficient indicates that specific schedules are associated with lower staff turnover rates, possibly due to greater job stability. The factor of having previously considered leaving a job due to moral distress (β = −0.315, *p* = 0.001) was one of the strongest negative predictors. Respondents whose workplaces use the TISS-28 tool showed significantly lower potential turnover scores. This result suggests that structured workload measurement can serve as a protective factor. Overall, these results confirm that both the organization of professional workload and subjective experiences of moral distress and burnout are directly related to staff resilience. The implementation of preventive mechanisms in practice, as well as structured workload measurement methods, such as TISS-28 or NAS, can be important tools in staff retention strategies.

To determine which psychosocial factors are associated with taking on additional duties and responsibilities in the work environment, a logistic regression analysis was performed. The dependent variable was binary: 0—has no additional duties, 1—has additional duties. The model initially classified all cases as “has duties” (classification accuracy: 63.2%). After including the independent variables, the classification accuracy increased to 66.5%, thereby improving the forecasting ability. The omnibus test yielded χ^2^ (3) = 17.069 (*p* < 0.001), indicating a significant improvement compared to the null model. Nagelkerke R^2^ = 0.143, which means that the model explains approximately 14.3% of the variation in the presence of additional duties. The sensitivity of the model (correctly identifying “has duties”) is 84.7%, while the specificity (correctly identifying “no duties”) is 35.1%. Of the three independent variables, moral distress total (MMD) appeared to be the only statistically significant predictor of MMD (β = −0.038, *p* < 0.001). With each increase in moral distress points, the likelihood that an employee will take on additional duties decreases, which could indicate a potential for distress overload and reduce the willingness or ability to perform additional tasks. Exp(B) = 0.963, which means that with each point increase in distress level, the odds of taking on additional duties decrease by 3.7%. The other two variables, CBI (*p* = 0.455) and intention to change jobs (*p* = 0.321), did not show a statistically significant effect.

To examine the interrelationships among the tools used in the study, including CBI, MMD, and AST, a correlation analysis was performed using both Pearson and Spearman correlation coefficients. The results reveal different strengths of association and statistical significance between the various factors. The correlation matrix is presented in Table 7.

There is a moderately strong and statistically significant positive correlation between moral distress and burnout, indicating that a higher level of moral distress is associated with a higher burnout rate. All three burnout subscales showed a moderately strong positive correlation with moral distress, which confirms the assumption that emotional tension and internal conflict arising in professional dilemmas are closely related to symptoms of burnout in all aspects—personal, professional, and in relationships with patients.

There is no significant correlation between either CBI or MMD and intention to leave a job, which may indicate that the intention to leave a job is the result of multiple factors, including work schedule, structured workload, or institutional culture, not just emotional exhaustion. The strength of these correlations is not sufficient to draw further conclusions about causal relationships; regression analysis is more appropriate for this purpose.

These findings demonstrate statistically significant positive correlations among moral distress, burnout, and staff turnover intention, which supports the hypothesis that psycho-emotional strain among ICU nurses is interconnected and may cumulatively influence their intention to leave the profession.

One-way analysis of variance (ANOVA) by region was performed to test whether the region of the respondents (0 = Riga, 1 = Vidzeme, 3 = Kurzeme, 4 = Zemgale) is statistically significantly related to CBI, staff turnover, and moral distress levels. To determine which specific regional groups differ significantly from one another, a post hoc Tukey HSD test was conducted. The test also included a Tukey HSD post hoc analysis to determine which groups differed from one another. Moral distress is the only one of the three indicators that differs significantly between regions. The ANOVA revealed a statistically significant difference in moral distress levels between regions, and the Tukey HSD post hoc test indicated that nurses in the Vidzeme region reported significantly lower levels of distress compared to those in Riga (*p* = 0.021) and Kurzeme (*p* = 0.034), while no significant difference was observed between Riga and Kurzeme. Burnout and staff turnover potential do not differ significantly, but trends indicate a potentially higher risk in Kurzeme and Riga.

To assess whether the intensity of workload perception in the last month is significantly related to burnout, personal burnout level, and moral distress, a one-way analysis of variance (ANOVA) was performed using four workload perception levels based on responses to the question about workload perception: from 1 (lowest) to 4 (highest). The results show that the ANOVA result (F = 8.754; *p* < 0.001) indicates a statistically significant difference between the groups. A post hoc Tukey HSD test was performed to explore pairwise differences. The results showed that burnout scores were significantly higher in group 3 (moderate-high workload) compared to group 2 (lower workload) (*p* = 0.017). The lowest average burnout level was among respondents with the lowest perceived workload (“2”—mean = 31.72), while the highest was among groups with a more intense perception (including “3” and “1”). The results of the ANOVA for moral distress (F = 5.635; *p* = 0.001) show statistically significant differences. The level of moral distress is lowest among respondents with a lower perceived workload (mean = 39.41) and increases significantly with a higher perceived workload (mean = 53.35 in group “4”).

Based on the in-depth quantitative analysis, it can be concluded that moral distress, burnout, and potential staff turnover among ICU nurses are multifactorial phenomena influenced by socio-demographic, professional, and subjective factors.

The level of moral distress is most significantly predicted by nurses’ previous experience with distress and their desire to leave the job, which shows a strong negative effect (β = –0.317; *p* < 0.001), indicating chronic accumulation of stress. In addition, educational level (β = 0.184; *p* = 0.043) and subjective perception of work overload (showing a significant trend, β = 0.132; *p* = 0.099) confirm that professional education and self-criticism may contribute to a higher perception of distress.

Taking on additional duties at work is inversely associated with moral distress—the higher the distress, the less likely the nurse is to take on additional responsibilities (β = −0.038; *p* < 0.001). This indicates a functional limitation resulting from moral distress, which may affect collective work efficiency.

Analysis of variance (ANOVA) by region, education, work schedule, and perceived workload reveals that moral distress and burnout differ significantly between regions and work organization models. It is particularly important that subjective workload perception correlates significantly with both increased burnout and moral distress, thus confirming the importance of emotional well-being in the work environment.

The results indicate that moral distress and burnout levels vary significantly across nurse demographics and ICU types, confirming the study’s objective to identify at-risk subgroups based on contextual and sociodemographic characteristics.

In summary, the results confirm that moral distress, burnout, and staff turnover risks are closely interrelated and significantly influenced by previous experience, subjective workload perception, and structural support (e.g., TISS-28 or NAS). The introduction of preventive mechanisms and the improvement of work organisation can serve as strategic orientations to strengthen professional resilience.

The findings of this study directly confirm the central hypotheses: moral distress and burnout are prevalent among ICU nurses; they are significantly interrelated; and these psychosocial factors substantially contribute to nurses’ intention to leave the profession. These results emphasize the urgent need for institutional strategies that address work overload, improve the ethical climate, and implement standardized workload assessment tools such as TISS-28 or NAS.

### 4.6. Hypothesis Testing

To test H1, which proposed that higher levels of moral distress would be associated with increased burnout among ICU nurses, a linear regression analysis was conducted. The results supported this hypothesis. Moral distress significantly predicted burnout (β = 0.545, *p* < 0.001). The model explained 29.7% of the variance in burnout scores (R^2^ = 0.297, F (1, 153) = 64.88, *p* < 0.001). This finding confirms that ICU nurses experiencing greater moral distress are more likely to report elevated levels of burnout.

For H2, which predicted that burnout would be positively associated with turnover intention, another linear regression analysis was conducted. The results also supported this hypothesis. Burnout was a significant predictor of turnover intention (β = 0.456, *p* < 0.001). The model accounted for 20.7% of the variance in turnover intention (R^2^ = 0.207, F(1,153) = 40.06, *p* < 0.001). These findings suggest that higher burnout levels among ICU nurses increase the likelihood that they will intend to leave their position.

Both regression models yielded statistically significant results, affirming the hypothesized relationships and providing empirical evidence for the proposed pathway from moral distress to burnout and subsequently to turnover intention.

To test H3, which proposed that burnout mediates the relationship between moral distress and turnover intention, a simple mediation model was tested using the PROCESS macro. In this model, moral distress was entered as the independent variable (X), burnout served as the mediator (M), and turnover intention served as the dependent variable (Y).

The overall model was statistically significant (R^2^ = 0.229, F (2, 152) = 22.64, *p* < 0.001). Moral distress was positively associated with burnout (a path: β = 0.545, SE = 0.068, t = 8.06, *p* < 0.001), and burnout was positively associated with turnover intention (b path: β = 0.337, SE = 0.072, t = 4.68, *p* < 0.001). The indirect effect of moral distress on turnover intention via burnout was β = 0.184, SE = 0.046, and the 95% bootstrap confidence interval did not include zero (CI [0.096, 0.280]), indicating a statistically significant mediation effect. The direct effect of moral distress on turnover intention (c’ path), controlling for burnout, remained significant but was reduced (β = 0.248, SE = 0.074, t = 3.36, *p* = 0.001), suggesting partial mediation.

These findings confirm that burnout partially mediates the relationship between moral distress and turnover intention among ICU nurses.

## 5. Discussion

The ICU environment is inherently demanding due to its high clinical acuity, frequent exposure to life-threatening situations, and ethical dilemmas, which together impose substantial psycho-emotional burdens on nurses. Consistent with international literature [17,45,46], this study confirms that moral distress and burnout are widespread and interrelated phenomena among Latvian ICU nurses. Our results support prior findings that emotional exhaustion and depersonalization are especially common in high-intensity settings, which correlate significantly with higher moral distress scores. This study confirms that these issues are prevalent among ICU nurses in Latvia, aligning with the international literature on the detrimental effects of persistent ethical and workload pressures [7,8,19].

Consistent with previous studies, our findings revealed moderate to high levels of moral distress among respondents, particularly in situations involving resource limitations and conflicting care expectations. Although the study design does not permit causal conclusions, the observed associations between moral distress and components of burnout (emotional exhaustion and depersonalization) support earlier findings that ethical dissonance contributes to psychological strain [19,46].

Burnout, as theorized by Maslach and Jackson [29], is a multifaceted syndrome characterized by emotional exhaustion, depersonalization, and reduced personal accomplishment. Our results confirm that Latvian ICU nurses experience moderate to high levels of burnout, with emotional exhaustion being the most pronounced. These findings are consistent with international evidence suggesting that ICU nurses are particularly vulnerable to burnout due to the emotionally intense nature of their work [18,46]. These findings also align with the JD-R model, which offers a broader theoretical lens for interpreting our results [30]. In interpreting these findings, the JD-R model offers a valuable framework. According to this model, burnout arises when job demands (e.g., high workload, emotional strain, ethical dilemmas) outweigh available job resources (e.g., team support, autonomy, institutional backing) [30]. In our sample, factors such as understaffing, long shifts, and the absence of psychological or ethical support reflect this imbalance, reinforcing the model’s applicability to Latvian ICU settings. The JD-R framework helps explain why, in environments with high demands and scarce resources, burnout and turnover intentions become systemic rather than individual issues, demanding organizational solutions [22,30].

The study also explored turnover intentions, revealing that nearly one in three ICU nurses had considered leaving their job. This corresponds with international data, with which similar psychological and systemic stressors have been linked to high nurse attrition rates [47,48]. Although our cross-sectional design does not establish causality, the strong correlations observed between burnout symptoms and turnover intentions suggest that emotional exhaustion may play a significant role in nurses’ decisions to leave their positions, as noted in prior studies [22,49].

From a theoretical standpoint, the phenomenon of moral distress challenges traditional ethical frameworks, which often assume that healthcare professionals can implement the most ethically sound solution. In practice, ICU nurses are frequently constrained by limited resources, rigid protocols, and hierarchical decision-making structures that limit their agency [50,51,52]. These systemic barriers contribute to ethical dissonance and subsequent distress. This dynamic is also captured by Karasek’s job demand–control model, in which high demands and low decision-making control contribute to chronic stress and burnout [52,53,54]. While Karasek’s model was not directly tested in our hypotheses, the low autonomy and limited decision-making authority reported by nurses in our sample support its relevance [35]. Future studies should explicitly incorporate this framework when exploring moral agency and systemic power asymmetries in ICU environments.

Despite its significance and empirical value, this study has several limitations that should be taken into account when interpreting the results and planning further research. The study used a voluntary sample, which may lead to bias in the sample. Survey participants were probably more motivated because they had already experienced moral distress or burnout and therefore wanted to express their opinion. This sampling peculiarity may affect the generalizability of the results to the whole population of Latvian ICU nurses.

The majority of respondents were from the Riga region, while no responses were received from Latgale. This limitation restricts the ability to compare regional differences and to fully reflect the situation in all healthcare regions of Latvia.

All data are based on respondents’ self-assessments, which may be subject to social desirability bias or memory errors. Moral distress, burnout, and staff turnover intentions can be sensitive topics where respondents choose to downplay or exaggerate their answers depending on perceived anonymity or fear of stigma.

As this was a cross-sectional study, it is not possible to draw conclusions about the causal relationship among moral distress, burnout, and staff turnover. A longitudinal study design would be necessary to interpret changes over time or cumulative effects more reasonably.

One key limitation of this study is the use of convenience sampling, which may introduce selection bias and limit the generalizability of the findings to the broader population of ICU nurses in Latvia. Despite attempts to include diverse hospital types and regions, the non-random nature of the sample may influence the representativeness of the results.

Intensity ratings can be deeply subjective and vary depending on the interpretation of the specific situation, the psychological state of the respondent at the time of the study, and their personal experiences. This means that emotional response indicators may not accurately reflect the objective severity of the situation but rather the intensity of perception. 

The study primarily focused on identifying risk factors; however, less attention was paid to possible protective mechanisms, such as management support, cooperation among colleagues, and the impact of professional training, all of which would be important for further intervention development.

## 6. Practical Implications

Importantly, our findings have practical implications for healthcare management and policy. Burnout and moral distress are not only personal or psychological issues but also organizational risks that compromise patient safety and care quality. Burnt-out nurses are more likely to make errors, show reduced compassion, and struggle with sustained attention, all of which have been associated with adverse patient outcomes, including infections, complications, and delayed interventions [22,55,56].

Furthermore, the cumulative effects of moral distress and burnout may lead to disengagement, reduced motivation to adhere to safety protocols, and emotional withdrawal from patients [57,58]. The link between nurse well-being and care quality is increasingly emphasized in the “Quadruple Aim” framework, which recognizes healthcare provider well-being as a critical element of system sustainability and patient safety [59].

Staff shortages, which are already a significant issue in Latvia, may be further exacerbated by burnout-driven turnover. OECD data highlight that Latvia has one of the lowest nurse-to-population ratios in Europe, a trend that has not improved in recent years [60]. Our study adds to this concern by showing that a considerable proportion of ICU nurses report intentions to leave their jobs, especially in response to moral distress and inadequate systemic support. Regional disparities, particularly the absence of responses from Latgale in our sample, further point to unequal healthcare access and workforce distribution.

Latvia’s ICU nurses face unique contextual stressors, including long shifts (24 h), limited psychological support, and the absence of structured workload assessment tools, such as the NAS or TISS-28 [61,62]. Our study supports earlier findings indicating that without standardized workload monitoring, hospitals may lack the data needed to justify staff increases or reorganize care processes [63,64]. ICU nurses often report feeling overwhelmed and “running all day without finishing their duties”, which exacerbates moral distress and undermines quality care.

Educational structures in Latvia also pose challenges. Despite the transition to a higher education model, nurse specialization in intensive care remains fragmented. Without standard training in ethical decision-making or communication skills for difficult situations, many nurses report feeling underprepared, potentially heightening their vulnerability to moral distress [65]. Moreover, the aging workforce—nearly 40% of Latvian nurses are over 55, which, combined with limited graduate retention, further threatens the system’s resilience [66].

This study offers valuable empirical insights into the interconnected challenges related to moral distress, burnout, and turnover intentions among ICU nurses in Latvia. By applying validated instruments across multiple centers, we identified significant psychological and organizational risks that echo international patterns while reflecting national specifics. Our recommendations are as follows:

1. Mandate standardized workload measurement tools (e.g., NAS) across ICUs to support staffing decisions and identify burnout risks.
2. Institutionalize access to psychological counseling and clinical ethics consultation as part of occupational health services.
3. Phase out 24 h shifts and establish evidence-based nurse–patient ratios to reduce physical and moral overload.
4. Enhance curricula with standardized modules on ethical decision-making, communication, and self-care strategies for critical care nurses.
5. Develop targeted retention programs and mentorship initiatives, especially for rural regions and younger professionals.

By adopting these actions, healthcare institutions and policymakers can address the root causes of ICU nurse burnout and contribute to the long-term sustainability and resilience of the Latvian healthcare system. Addressing these factors is essential not only for protecting the mental health of ICU nurses but also for ensuring patient safety and sustaining the healthcare system in Latvia. Future research should adopt longitudinal designs, include underrepresented regions, and explore institutional resilience factors that could mitigate the identified risks.

## 7. Future Research

Given the cross-sectional design of this study and its inherent limitations, future research should adopt longitudinal approaches to examine causal pathways and the long-term effects of moral distress and burnout on ICU nurses’ well-being and professional outcomes. Tracking changes over time would help identify whether these phenomena are transient reactions to acute stressors or reflect chronic systemic dysfunction [24,46].

Moreover, qualitative studies are needed to explore the lived experiences of ICU nurses in depth, especially regarding moral conflict, professional identity, and coping strategies. Such insights could complement quantitative findings and help develop more tailored interventions [8,24,50].

Future research should also aim to include underrepresented geographic areas, particularly Latgale, to improve the generalizability and regional representativeness of findings. Comparative studies between urban and rural ICUs could shed light on structural inequalities in staff support, workload distribution, and moral agency [47,48].

Given the significant role of perceived organizational support and team dynamics observed in other healthcare systems [28,45,54], future studies in Latvia should examine how institutional factors such as leadership, ethics infrastructure, and communication culture mediate the impact of high job demands.

Finally, intervention studies should be prioritized to test the effectiveness of specific strategies, such as clinical ethics consultation services, workload monitoring tools (e.g., NAS), or psychological resilience training, in reducing distress and preventing burnout. Such trials would provide a much-needed evidence base for national-level policy reforms and institutional practices [64].

## 8. Conclusions

The results of this study indicate the psychosocial risks in the professional environment of Latvian ICU nurses, confirming that moral distress and burnout are significant factors affecting the well-being and retention of these specialists in their profession. The results show that ICU nurses regularly encounter morally and ethically challenging situations, which not only recur with high frequency but also cause significant psychological strain. A two-dimensional MMD-HP scale analysis showed that certain clinical situations, such as excessive documentation, care without clear treatment goals, or actions contrary to personal values, are perceived as particularly distressing and emotionally burdensome.

The CBI scale scores confirmed a moderately high level of burnout among ICU nurses, especially in the personal and work-related burnout dimensions. All three subscales showed a strong risk of psychological overload, while client-related burnout was comparatively lower, indicating professional competence in managing patient care, but at the same time pointing to structural problems in the organization and work environment. The results of potential staff turnover revealed high variability among respondents; however, psychometric analysis suggested that this scale should be improved to ensure greater convergence between its items.

Multifactorial regression analysis identified the main risk factors for moral distress, burnout, and intentions to change jobs. The strongest predictor for all three manifestations was related to respondents’ previous or current desire to leave their job due to moral distress. This indicates a possible cumulative or chronic emotional impact, which over time contributes to emotional exhaustion and results in professional disengagement. In addition, higher levels of education and increased subjective workload perception were also associated with higher levels of distress and burnout. Marital status, as well as having completed a distress scale in the past, was correlated with an increased risk of burnout, possibly reflecting heightened emotional sensitivity or previous experience of stress.

Logistic regression revealed that a higher level of moral distress reduces the likelihood that the nurse will take on additional duties, indicating a loss of motivation and depletion of coping resources. This finding is an important signal to management about the potential consequences at the professional engagement level. The ANOVA results confirmed that regional differences in distress experience exist and that a higher subjective perception of workload is associated with both increased distress and burnout levels.

Finally, correlation analysis demonstrated a statistically significant and moderately strong positive association between moral distress and burnout, which is consistent with international data. This link highlights the need to develop comprehensive prevention systems that simultaneously address both ethical and emotional risk factors.

Based on the study findings, we recommend implementing structured workload monitoring tools (e.g., NAS, TISS-28), integrating regular psychological resilience training, and establishing early warning systems for burnout and moral distress. Health policymakers are encouraged to incorporate psycho-emotional well-being indicators into national nursing workforce strategies. Moreover, professional education programs should include modules on ethical conflict resolution and emotional self-regulation to strengthen nurses’ long-term resilience.

In summary, the results of this study provide an empirical basis for strategies aimed at reducing moral distress, preventing burnout, and strengthening staff resilience in Latvian ICU practice. The psychometric quality of the scales confirms their suitability for further research and clinical use. These findings offer a solid foundation for long-term improvements in workforce policy, institutional standards, and targeted support systems for ICU nurses.

## Figures and Tables

**Table 1:** Summary of top 5 MMD-HP frequency items with the highest item-total correlation and frequency mean.

Items	Mean	SD	Corrected Item-Total Correlation	Squared Multiple Correlation	Cronbach’s Alpha if Ited Deleted
2. Follow the family’s insistence to continue aggressive treatment even though I believe it is not in the best interest of the patient.	1.82	1.271	0.677	0.775	0.934
5. Continue to provide aggressive treatment for a person who is most likely going to die, regardless of this treatment, when no one will make a decision to withdraw it.	2.35	1.287	0.599	0.682	0.935
10. Follow a physician or family member’s request not to discuss the patient’s prognosis with the patient/family.	2.11	1.614	0.160	0.285	0.943
16. Be required to care for more patients than I can safely care for.	2.18	1.346	0.587	0.646	0.936
19. Have excessive documentation requirements that compromise patient care.	2.26	1.333	0.496	0.590	0.937

**Table 2:** Summary of top 5 MMD-HP frequency items with the highest item-total correlation and intensity mean.

Items	Mean	SD	Corrected Item-Total Correlation	Squared Multiple Correlation	Cronbach’s Alpha if Ited Deleted
5. Continue to provide aggressive treatment for a person who is most likely going to die, regardless of this treatment, when no one will make a decision to withdraw it.	2.45	1.456	0.734	0.839	0.975
8. Participate in care that causes unnecessary suffering or does not adequately relieve pain or symptoms.	2.52	1.316	0.823	0.814	0.975
16. Be required to care for more patients than I can safely care for.	2.63	1.285	0.802	0.815	0.975
17. Experience compromised patient care due to lack of resources/equipment/bed capacity.	2.33	1.275	0.671	0.686	0.976
19. Have excessive documentation requirements that compromise patient care.	2.34	1.292	0.774	0.859	0.975

**Table 3:** Copenhagen Burnout Inventory results.

Items	Cronbach’s Alpha	Mean	SD	Corrected Item-Total Correlation	Squared Multiple Correlation	Cronbach’s Alpha if Ited Deleted
1. How often do you feel tired?	0.894 (PRB)	2.35	0.762	0.630	0.795	0.925
2. How often are you physically exhausted?		2.35	0.779	0.667	0.780	0.924
5. How often do you feel worn out?		2.65	0.691	0.675	0.608	0.924
7. Do you feel worn out at the end of the working day?	0.755 (WRB)	2.86	0.886	0.555	0.571	0.926
11. Is your work emotionally exhausting?		2.39	1.003	0.720	0.728	0.922

**Table 4:** Potential staff turnover scale—top 5 items with highest mean scores.

Items	Mean	SD	Corrected Item-Total Correlation	Squared Multiple Correlation	Cronbach’s Alpha if Ited Deleted
1. I intend to stay in my current job for some time.	4.65	1.719	0.145	0.577	0.314
7. I have been at this workplace for as long as I want to be.	4.67	1.668	0.177	0.639	0.304
8. I am sure that I will stay here for a while.	4.34	1.919	0.052	0.764	0.346
9. I have no specific idea how much longer I will stay here.	4.53	1.828	0.239	0.380	0.279
10. I intend to keep my job at this organization for some time.	4.43	1.848	0.084	0.396	0.335

**Table 5:** Statistically significant CBI predictors.

Predictor	Beta	*p*-Value	VIF
Family status	0.175	0.01	1.165
Fulfilling the MMD-HP	−0.241	0.002	1.536
Have you ever left or considered leaving clinical work due to moral distress?	−0.383	0.0	1.828
Are you currently considering leaving your position due to moral distress?	−0.387	0.0	1.614

**Table 6:** Statistically significant predictors of the potential staff turnover scale (*p* < 0.005).

Predictor	Beta	*p*-Value	VIF
Working hours	−0.184	0.032	1.462
Have you ever left or considered leaving clinical work due to moral distress?	−0.315	0.001	1.828
In your opinion, are preventive measures necessary to address or reduce the factors contributing to burnout?	+0.466	<0.001	3.394
Are you required to complete the TISS-28 instrument in your workplace?	−1.109	0.012	38.289

**Table 7:** Pearson and Spearman correlation matrix among burnout, moral distress, and turnover intention scales.

		CBI	AST	MMD-HP
CBI	Pearson	1	0.050	0.357
	Sig.		0.536	<0.001
	Spearman’s Rho	1.000	0.117	0.406
	Sig.		0.148	<0.001
AST	Pearson	0.050	1	0.095
	Sig.	0.536		0.238
	Spearman’s Rho	0.117	1.000	0.125
	Sig.	0.148		0.123
MMD-HP	Pearson	0.357	0.095	1
	Sig.	<0.001	0.238	
	Spearman’s Rho	0.406	0.125	1.000
	Sig.	<0.001	0.123	

## Data Availability

The datasets produced and examined in this study can be obtained from the corresponding author upon a reasonable request. All data generated or analyzed during this study are provided, within the published article. The data utilized in this study are confidential.

## Supplementary Materials

The following supporting information can be downloaded at: https://www.mdpi.com/article/10.3390/ijerph22081261/s1, Table S1: Demographic data of respondents.; Table S2: Results of the moral distress scale (frequency).; Table S3: Results of the moral distress scale (intensity).; Table S4: Copenhagen Burnout Inventory results.; Table S5: Potential staff turnover scale results.

## Abbreviations

ICU	Intensive Care Units
WHO	World Health Organization
TISS-28	Therapeutic Intervention Scoring System—28 items
NAS	The Nursing Activities Score
H1	Hypothesis one
H2	Hypothesis two
H3	Hypothesis three
JD-R	Job Demands–Resources
COVID-19	Coronavirus Disease 2019
MMD-HP	Moral Distress Scale for Healthcare Professionals
CBI	Copenhagen Burnout Inventory
ATS	Anticipated Turnover Scale
PĒK	Pētījuma Ētikas komitēja
CMB	Common Method Bias
EFA	Exploratory Factor Analysis
PCA	principal component analysis
IBM SPSS	Statistical Package for the Social Sciences
r	Pearson Correlation Coefficient
ρ	Spearmon Correlation Coefficient
ANOVA	Analysis of Variance
VIF	Variance Inflation Factor
SD	Standard Deviation
h	Hours
M	Mean
S.E.	Standard Error
PRB	Personal-related burnout
WRB	Work-related burnout
CRB	Client-related burnout
R and R^2^	Determination coefficient
F	Regression model
Tukey HSD	Tukey Honestly Significant Difference
ICN	International Council of Nurses
OECD	Organisation for Economic Co-operation and Development
