# Supplementary material for: Moral Distress, Professional Burnout, and Potential Staff Turnover in Intensive Care Nursing Practice in Latvia—Phase 1
Source: Int J Environ Res Public Health. 2025 Aug 12;22(8):1261. doi: 10.3390/ijerph22081261 (PMC12386003; doi:10.3390/ijerph22081261)
Supplement: Supplementary file 1 [file ijerph-22-01261-s001.zip › ijerph-3766916-supplementary.pdf]

Suppementary Materials

Table S1. Demographic data of respondents.

Question	n=155
Gender	Female—149 (96,1%)<br>Male—2 (1.3%)<br>Do not wish to specify—4 (2,6%)
Region of workplace	Riga region—123 (79,4%)<br>Kurzeme region—26 (16,8%)<br>Latgale region—0 (0%)<br>Vidzeme region—2 (1,3%)<br>Zemgale region—4 (2,6%)
Family status	Married—78 (50,3%)<br>Not married – 31 (20,00)<br>Single—1 (0,6%)<br>Divorced—23 (14.8%)<br>Widowed—10 (6,5%)<br>Cohabiting—12 (7,7%)
Education	Vocational secondary education—29 (18,7%)<br>First level professional higher education—32 (20,6%)<br>Second level professional higher education or Bachelor's degree—73 (47,1%)<br>Master's degree—20 (12,9%)<br>Doctoral degree—1 (0.6%)
Position	Medical assistant—10 (6,5%)<br>Nurse—126 (81.3%)<br>Physician assistant—8 (5,2%)<br>Head nurse—9 (5,8%)<br>Deputy head nurse—2 (1,3%)
Department profile	General ICU—134 (86,5%)<br>Neonatal ICU—5 (3,2%)<br>Pediatric ICU-3 (1,9%)<br>Cardiac ICU—10 (6,5%)<br>Toxicology and Sepsis ICU – 3 (1,9%)
Total workload	Up to 0.25 workload (10 h per week)—4 (2,6%)<br>0.25–0.5 workload (10–20 h per week)—8 (5,2%)<br>0.5–0.75 workload (20–30 h per week)—7 (4,5%)<br>0.75–1.0 workload (30–40 h per week)—23 (14,8%)<br>1.0–1.25 h (40–50 h per week)— 78 (50,3%)<br>1.25–1.5 h (50–60 h per week)—23 (14,8%)<br>More than 1,5 (more than 60 h per week) -12 (7,7%)
Working hours	Daily work—12 (7,7%)<br>Shift work—98 (63,2%)<br>Both daily and shift work—45 (29,0%)
Number of workplaces	1 job – 88 (56,8%)<br>2 jobs – 59 (38,1%)

	3 jobs – 6 (3,9%)<br>4 jobs – 2 (1,3%)
Additional responsibilities	Yes – 57 (36,8%)<br>No – 98 (63,2%)
Workload in the last month	Low—6 (3,9%)<br>Moderate—58 (37,4%)<br>High—65 (41,9)<br>Very high—26 (16,8%)
Fulfilling the MMD-HP (previous)	Yes –34 (21,9%)<br>No – 121 (78,1%)
Fulfilling the CBI (previous)	Yes – 99 (63,9%)<br>No – 56 (36,1)
Have you ever left or considered leaving clinical work due to moral distress?	Maybe -78 (50,3%)<br>Yes – 10 (6,5%)<br>No – 67 (43,2%)
Are you currently considering leaving your position due to moral distress?	Yes – 39 (25,2%)<br>No – 116 (74,8%)
In your opinion, are preventive measures necessary to address or reduce the causes of moral distress?	Yes – 140 (90,3%)<br>No – 15 (9,7%)
In your opinion, are preventive measures necessary to address or reduce the factors contributing to burnout?	Yes – 146 (94,2%)<br>No – 9 (5,8%)
Is the TISS-28 instrument implemented in your workplace?	Yes – 114 (73,5%)<br>No – 4 (2,6%)<br>I don't know what TISS-28 is – 37 (23,9%)
Are you required to complete the TISS-28 instrument in your workplace?	Yes – 114 (73,5%)<br>No – 8 (5,2%)<br>I don't know what TISS-28 is – 33 (21,3%)

Table S2. Results of the moral distress scale (frequency).

Items	Mean	SD	S.E.	Scale Mean if Item Deleted	Scale Variance if Item Deleted	Corrected Item-Total Correlation	Squared Multiple Correlation	Cronbach's Alpha if Ited Deleted
1. Witness healthcare providers giving "false hope" to a patient or family.	1,35	1,067	0,086	35,45	333,288	0,571	0,719	0,936
2.Follow the family's insistence to continue aggressive treatment even though I believe it is not in the best interest of the patient.	1,82	1,271	0,102	34,99	324,104	0,677	0,775	0,934
3.Feel pressured to order or carry out orders for what I consider to be unnecessary or inappropriate tests and treatments.	1,63	1,271	0,102	35,18	328,915	0,659	0,728	0,935
4.Be unable to provide optimal care due to pressures from administrators or insurers to reduce costs.	1,44	1,134	0,091	35,37	330,753	0,598	0,604	0,935

5.Continue to provide aggressive treatment for a person who is most likely to die regardless of this treatment when no one will make a decision to withdraw it.	2,35	1,287	0,103	34,46	327,133	0,599	0,682	0,935
6.Be pressured to avoid taking ATSion when I learn that a physician, nurse, or other team colleague has made a medical error and does not report it.	0,70	0,920	0,074	36,10	336,885	0,562	0,595	0,936
7.Be required to care for patients whom I do not feel qualified to care for.	0,92	0,990	0,080	35,88	332,753	0,636	0,672	0,935
8.Participate in care that causes unnecessary suffering or does not adequately relieve pain or symptoms.	1,40	1,182	0,095	35,41	327,373	0,653	0,697	0,935
9.Watch patient care suffer because of a lack of provider continuity.	1,41	1,109	0,089	35,40	334,216	0,524	0,582	0,936
10.Follow a physician's or family member's request not to discuss the patient's prognosis with the patient/family.	2,11	1,614	0,130	34,70	344,486	0,160	0,285	0,943
11.Witness a violation of a standard of prATSice or a code of ethics and not feel sufficiently supported to report the violation.	1,05	0,996	0,080	35,76	338,118	0,480	0,608	0,937
12.Participate in care that I do not agree with, but do so because of fears of litigation.	0,62	0,975	0,078	36,19	335,439	0,568	0,572	0,936
13.Be required to work with other healthcare team members who are not as competent as patient care requires.	1,63	1,218	0,098	35,18	325,006	0,688	0,719	0,934
14.Witness low quality of patient care due to poor team communication.	1,54	1,186	0,095	35,26	325,248	0,703	0,710	0,934
15.Feel pressured to ignore situations in which patients have not been given adequate information to ensure informed consent.	0,86	0,074	0,926	35,94	336,315	0,575	0,616	0,936
16.Be required to care for more patients than I can safely care for.	2,18	1,346	0,108	34,63	326,366	0,587	0,646	0,936
17.Experience compromised patient care due to lack of resources/equipment/bed capacity.	1,57	1,087	0,087	35,24	330,183	0,641	0,747	0,935
18.Experience lack of administrative ATSion or support for a problem that is compromising patient care.	1,59	1,121	0,090	35,22	329,627	0,634	0,722	0,935
19.Have excessive documentation requirements that compromise patient care.	2,26	1,333	0,107	34,55	330,873	0,496	0,590	0,937
20.Fear retribution if I speak up.	1,35	1,204	0,097	35,46	326,717	0,656	0,800	0,935
21.Feel unsafe/bullied amongst my own colleagues.	0,78	0,989	0,079	36,03	337,376	0,505	0,702	0,936
22.Be required to work with abusive patients/family members who are compromising quality of care.	1,53	1,089	0,087	35,28	333,267	0,560	0,490	0,936
23.Feel required to overemphasize tasks and productivity or quality measures at the expense of patient care.	1,12	1,050	0,084	35,69	329,761	0,677	0,679	0,934
24.Be required to care for patients who have unclear or inconsistent treatment plans or who lack goals of care.	1,26	1,018	0,082	35,55	327,353	0,768	0,673	0,933
25.Work within power hierarchies in teams, units, and my institution that compromise patient care.	0,92	1,044	1,044	35,89	332,007	0,620	0,791	0,935

26.Participate on a team that gives inconsistent messages to a patient/family.	0,57	0,919	0,074	36,24	334,079	0,648	0,795	0,935
27.Work with team members who do not treat vulnerable or stigmatized patients with dignity and respect.	0,87	1,085	0,087	35,94	334,502	0,530	0,644	0,936

Table S3. Results of the moral distress scale (intensity).

Items	Mean	SD	S.E.	Scale Mean if Item Deleted	Scale Variance if Item Deleted	Corrected Item-Total Correlation	Squared Multiple Correlation	Cronbach's Alpha if Ited Deleted
1. Witness healthcare providers giving "false hope" to a patient or family.	2,03	1,446	0,116	55,40	764,703	0,747	0,871	0,975
2.Follow the family's insistence to continue aggressive treatment even though I believe it is not in the best interest of the patient.	2,28	1,375	0,110	55,15	771,244	0,691	0,851	0,976
3.Feel pressured to order or carry out orders for what I consider to be unnecessary or inappropriate tests and treatments.	2,17	1,371	0,110	55,26	763,050	0,804	0,887	0,975
4.Be unable to provide optimal care due to pressures from administrators or insurers to reduce costs.	2,20	1,398	0,112	55,23	765,228	0,759	0,840	0,975
5.Continue to provide aggressive treatment for a person who is most likely to die regardless of this treatment when no one will make a decision to withdraw it.	2,45	1,456	0,117	54,98	764,707	0,734	0,839	0,975
6.Be pressured to avoid taking ATSion when I learn that a physician, nurse, or other team colleague has made a medical error and does not report it.	2,28	1,413	0,114	55,14	758,382	0,842	0,858	0,975
7.Be required to care for patients whom I do not feel qualified to care for.	2,15	1,373	0,110	55,27	764,549	0,783	0,805	0,975
8.Participate in care that causes unnecessary suffering or does not adequately relieve pain or symptoms.	2,52	1,316	0,106	54,90	764,283	0,823	0,814	0,975
9.Watch patient care suffer because of a lack of provider continuity.	2,32	1,263	0,101	55,11	765,631	0,839	0,826	0,975
10.Follow a physician's or family member's request not to discuss the patient's prognosis with the patient/family.	1,72	1,449	0,116	55,71	773,870	0,619	0,674	0,976
11.Witness a violation of a standard of practice or a code of ethics and not feel sufficiently supported to report the violation.	2,15	1,368	0,110	55,27	759,913	0,850	0,890	0,975
12.Participate in care that I do not agree with, but do so because of fears of litigation.	1,99	1,430	0,115	55,43	760,169	0,807	0,866	0,975
13.Be required to work with other healthcare team members who are not as competent as patient care requires.	2,21	1,220	0,098	55,22	774,848	0,729	0,831	0,975
14.Witness low quality of patient care due to poor team communication.	2,21	1,273	0,102	55,22	766,042	0,826	0,874	0,975
15.Feel pressured to ignore situations in which patients have not been given adequate information to ensure informed consent.	2,17	1,313	0,105	55,23	765,829	0,802	0,815	0,975
16.Be required to care for more patients than I can safely care for.	2,63	1,285	0,103	55,26	765,829	0,802	0,815	0,975
17.Experience compromised patient care	2,33	1,275	0,102	54,80	776,213	0,671	0,686	0,976

due to lack of resources/equipment/bed capacity.								
18.Experience lack of administrative action or support for a problem that is compromising patient care.	2,25	1,251	0,101	55,10	768,374	0,791	0,890	0,975
19.Have excessive documentation requirements that compromise patient care.	2,34	1,292	0,104	55,17	770,560	0,774	0,859	0,975
20.Fear retribution if I speak up.	1,74	1,372	0,110	55,08	784,999	0,542	0,757	0,976
21.Feel unsafe/bullied amongst my own colleagues.	1,54	1,465	0,118	55,68	767,880	0,738	0,799	0,975
22.Be required to work with abusive patients/family members who are compromising quality of care.	2,17	1,460	0,117	55,88	766,818	0,702	0,751	0,976
23.Feel required to overemphasize tasks and productivity or quality measures at the expense of patient care.	1,98	1,312	0,105	55,25	765,813	0,717	0,771	0,975
24.Be required to care for patients who have unclear or inconsistent treatment plans or who lack goals of care.	2,02	1,253	0,101	55,45	763,651	0,835	0,886	0,975
25.Work within power hierarchies in teams, units, and my institution that compromise patient care.	1,84	1,356	0,109	55,41	764,918	0,855	0,904	0,975
26.Participate on a team that gives inconsistent messages to a patient/family.	1,73	1,355	0,109	55,59	7630776	0,804	0,884	0,975
27.Work with team members who do not treat vulnerable or stigmatized patients with dignity and respect.	2,01	1,437	0,115	55,41	758,712	0,822	0,875	0,975

Table S4. Copenhagen Burnout Inventory results.

Items	Cronbach's Alpha	Mean	SD	S.E.	Scale Mean if Item Deleted	Scale Variance if Item Deleted	Corrected Item-Total Correlation	Squared Multiple Correlation	Cronbach's Alpha if Ited Deleted
1.How often do you feel tired?	0,894 (PRB)	2,35	0,762	0,061	35,24	131,114	0,630	0,795	0,925
2.How often are you physically exhausted?		2,35	0,779	0,063	35,24	130,261	0,667	0,780	0,924
3.How often are you emotionally exhausted?		2,34	0,871	0,070	35,25	128,086	0,704	0,734	0,923
4.How often do you think: "I can't take it any more"?		1,90	1,005	0,081	35,69	124,241	0,780	0,770	0,921
5.How often do you feel worn out?		2,65	0,691	0,055	34,95	131,543	0,675	0,608	0,924
6.How often do you feel weak and susceptible to illness?		1,79	1,061	0,085	35,80	127,797	0,575	0,618	0,926
7.Do you feel worn out at the end of the working day?	0,755 (WRB)	2,86	0,886	0,071	34,74	130,702	0,555	0,571	0,926
8.Are you exhausted in the morning at the thought of another day at work?		1,96	1,104	0,089	35,63	122,766	0,766	0,716	0,921
9.Do you feel that every working hour is tiring for you?		1,74	1,037	0,083	35,85	123,244	0,799	0,745	0,920
10.Do you have enough energy for family and friends during leisure tim		2,20	0,878	0,071	35,39	155,708	-0,628	0,502	0,947
11.Is your work emotionally exhausting?		2,39	1,003	0,081	35,21	125,542	0,720	0,728	0,922
12.Does your work frustrate you?		1,55	0,988	0,079	36,05	125,199	0,748	0,753	0,922
13.Do you feel burnt out because of your work?		1,79	1,075	0,086	35,81	123,131	0,773	0,789	0,921
14.Do you find it hard to work with clients?	0,894 (CRB)	1,33	0,774	0,062	36,26	129,793	0,699	0,714	0,923

15.Does it drain your energy to work with clients?	1,66	0,841	0,068	35,94	128,217	0,725	0,738	0,923
16.Do you find it frustrating to work with clients?	1,34	0,862	0,069	36,26	129,323	0,645	0,691	0,924
17.Do you feel that you give more than you get back when you work with clients?	2,19	1,140	0,092	35,40	125,917	0,606	0,563	0,925
18.Are you tired of working with clients?	1,65	0,944	0,076	35,94	127,912	0,652	0,771	0,924
19.Do you sometimes wonder how long you will be able to continue working with clients?	1,55	1,191	0,096	36,05	122,978	0,694	0,783	0,923

Table S5. Potential staff turnover scale results.

Items	Mean	SD	S.E.	Scale Mean if Item Deleted	Scale Variance if Item Deleted	Corrected Item-Total Correlation	Squared Multiple Correlation	Cronbach's Alpha if Ited Deleted
1.I intend to stay in my current job for some time.	4,65	1,719	0,138	37,95	63,270	0,145	0,577	0,314
2.I am almost certain that I will leave my job in the near future.	2,26	2,111	0,170	40,34	59,211	0,201	0,785	0,288
3.Deciding whether to stay or leave my job is not an essential issue for me at this time.	4,38	1,938	0,156	38,22	57,705	0,297	0,437	0,523
4.I have already made the decision to stay with or leave this organization in the short term.	2,60	2,244	0,180	40,00	60,039	0,148	0,691	0,310
5.If I were to receive another job offer tomorrow, I would seriously consider it.	3,12	2,178	0,175	39,48	61,212	0,125	0,579	0,320
6.I have no intention of leaving my current job.	3,63	2,274	0,183	38,97	66,973	-0,052	0,713	0,394
7.I have been at this workplace for as long as I want to be.	4,67	1,668	0,134	37,93	62,755	0,177	0,639	0,304
8.I am sure that I will stay here for a while.	4,34	1,919	0,154	38,26	64,907	0,052	0,764	0,346
9.I have no specific idea how much longer I will stay here.	4,53	1,828	0,147	38,07	60,092	0,239	0,380	0,279
10.I intend to keep my job at this organization for some time.	4,43	1,848	0,148	38,17	64,309	0,084	0,396	0,335
11.I have major doubts about whether or not I will stay in this organization.	2,18	2,140	0,172	40,42	65,868	-0,007	0,825	0,372
12.I plan to leave this job soon.	1,80	2,062	0,166	40,80	63,849	0,064	0,805	0,343
